# Harnessing Oncolytic Virus-Mediated Anti-Tumor Immunity

**DOI:** 10.3389/fonc.2014.00337

**Published:** 2014-11-24

**Authors:** Volker Schirrmacher, Philippe Fournier

**Affiliations:** ^1^DKFZ, Heidelberg, Germany; ^2^IOZK, Cologne, Germany

**Keywords:** oncolytic virus, anti-tumor activity, tumor-associated antigen, oncolytic virotherapy, immunovirotherapy, immunotherapeutic approaches, anti-viral response

Oncolytic viruses (OVs) selectively infect, replicate in, and kill tumor cells. For a long time, the therapeutic efficacy of OVs was thought to depend mainly on this mechanism of direct viral oncolysis. Nowadays, however, the post-oncolytic anti-tumor activity induced by the OV therapy is considered a key factor for an efficient therapeutic activity. The research topic addresses these issues and discusses future strategies how to further optimize OVs anti-tumor activity.

The first two articles deal with viral oncolysis and the immune response. Guo et al. (1) from the University of Pittsburgh Cancer Institute (USA) point out that dying the right way is a key to eliciting potent anti-tumor immunity. They describe that OVs induce mostly immunogenic cancer cell death (ICD) including immunogenic apoptosis, necrosis/necroptosis, pyroptosis, and autophagic cell death. A review of recent advances in our understanding of danger signals is followed by a discussion of potential combination strategies to target cells into specific modes of ICD. Thorne (2) from the same Institution argues in his perspective article that the immune response raised by an OV can also hinder optimal therapeutic activity and repeat dosing. Using oncolytic vaccinia virus (VV) as an example, Thorne summarizes approaches to enhance the anti-tumor immune response by the introduction of immune stimulatory transgenes. His article points our attention also toward interesting new alternative strategies.

The next four articles review and discuss in more detail post-oncolytic anti-tumor immune responses. Gujar and Lee (3) from the Dalhousie University of Halifax (Canada) discuss how OV-induced immunological events override tumor-associated antigen (TAA) presentation impairment and promote appropriate T cell interaction with antigen-presenting cells (APC). Woller et al. (4) from the Medical School in Hannover (Germany) review the role of viral oncolysis for induction of ICD including autophagy, DAMPs and PAMPs, and the ER–stress response. Finally, they highlight developments for exploiting the vaccinative potential of oncolytic virotherapy. Moehler et al. (5) from the University Medical Center in Mainz (Germany) together with Jean Rommelaere from the DKFZ, Heidelberg (Germany) draw our attention to oncolytic parvoviruses and review their evidence that these can trigger maturation of dendritic cells (DCs) and induce activation of antigen-specific cytotoxic T cells. Finally, they discuss the clinical potential of the immunovirotherapy concept and its combination with new targeted therapies or with immune checkpoint blocking antibodies. Janelle and Lamarre (6) from the INRS-Institut Armand-Frappier in Quebec (Canada) discuss the question of how to assess anti-tumor immunity. They exemplify this by reviewing experimental studies with B16 mouse melanoma, which is treated by vesicular stomatitis virus (VSV) variants.

How can OVs be harnessed or combined with other agents such as antibodies to mediate stronger anti-tumor effects? This question is discussed by the following two manuscripts. Bauzon and Hermiston (7) from the Bayer HealthCare US Innovation Center in San Francisco (USA) propose to merge OVs with immune checkpoint blocking antibodies. Immune checkpoints refer to a number of inhibitory pathways that play crucial roles in maintaining self-tolerance and immune homeostasis. The discovery and targeting of immune checkpoints has opened a new immunotherapeutic avenue generating very promising clinical results. Arguments are put forward to combine this strategy with an OV therapy to create synergies between both approaches. This might result in enhanced safety and efficacy and would be also economically advantageous. Schirrmacher and Fournier (8) from the DKFZ, Heidelberg (Germany) and from the IOZK in Cologne (Germany) put forward in a perspective article a new concept of a multimodal cancer therapy involving oncolytic Newcastle disease virus (NDV), autologous immune cells (activated T cells and/or polarized DC1), and bi-specific antibodies (bsAbs). The bsAbs they created are NDV-specific single-chain (scFv) antibodies fused with anti-CD3 or anti-CD28 T cell activating scFvs. These reagents, upon attachment to NDV infected tumor cells, are reported to have a strong potential to activate cancer patients T cells, including TAA-specific memory T cells and not TAA-specific naïve T cells. Such *ex vivo* activated autologous T cells can be transferred back to the patient. To increase their tumor targeting efficacy, it is suggested to pre-activate the tumor microenvironment by low dose irradiation or by local hyperthermia. Tumor targeting of grafted T cells is suggested to become also improved via cell-bound tri-specific antibodies targeting a tumor introduced viral antigen such as HN of NDV.

Delivery of OVs is another important aspect for achievement of optimal effects. Tai and Auer (9) from the Ottawa Hospital Research Institute, Ottawa (Canada) argue that the optimal time point should be either pre- or post-operative to counteract surgery induced immunosuppression and to attack post-operative metastases. They review their preclinical surgery models, in which pre-operative OVs prevented post-operative NK cell dysfunction and attenuated tumor dissemination. Altomonte and Ebert (10) from the Klinikum rechts der Isar, Munich (Germany) discuss the particular challenges of OV therapy for hepatocellular carcinoma as well as some potential strategies for modulating the immune system and synergizing it with the hepatic microenvironment. Combination strategies involving the adoptive transfer of immune cells together with OVs are expected as an exciting new approach.

Successful therapy using OVs will ultimately depend on effectively navigating the delicate balance between the anti-viral response and the anti-tumor immune response such as to minimize the former in the short term and maximize the latter in the long term. As outlined by Forbes et al. (11) from the Ottawa Hospital Research Institute, Ottawa (Canada), several approved drugs and novel small molecules can be effective tools to dampen the innate and adaptive anti-viral responses, increase the anti-tumor immune response, or both. Such approaches are discussed to be undoubtedly context dependent (e.g., tumor type and tumor site) and OV-dependent. This topic of combining oncolytic virotherapy with chemotherapy is further discussed by Nguyen et al. (12) from the McMaster University, Hamilton (Canada). With a particular focus on pharmaceutical immunomodulators they discuss how specific therapeutic contexts may alter the effects of these synergistic combinations and their implications for future clinical use.

It is remarkable to what extent experts from Canada, Germany, and the USA are in accord in this e-book by emphasizing the potential importance of OVs on systemic T cell-mediated anti-tumor immunity.

## Conflict of Interest Statement

The author declares that the research was conducted in the absence of any commercial or financial relationships that could be construed as a potential conflict of interest.
